# Investigating multimorbidity patterns and associated risk factors in the fasa adults cohort study (FACS): A latent class analysis

**DOI:** 10.1371/journal.pone.0335177

**Published:** 2025-11-03

**Authors:** Mehdi Sharafi, Najibullah Baeradeh, Mohammad Ali Mohsenpour, Sima Afrashteh, Pezhman Bagheri, Omid Keshavarzian, Mohammad Amin Annabi, Fatemeh Nekouei, Mojtaba Farjam

**Affiliations:** 1 Cellular and Molecular Research Center, Gerash University of Medical Sciences, Gerash, Iran; 2 Noncommunicable Diseases Research Center, Fasa University of Medical Sciences, Fasa, Iran; 3 Department of Public Health, Ferdows Faculty of Medical Sciences, Birjand University of Medical Sciences, Birjand, Iran; 4 Nutrition Research Center, Shiraz University of Medical Sciences, Shiraz, Iran; 5 Department of Biostatistics and Epidemiology, Faculty of Health and Nutrition, Bushehr University of Medical Sciences, Bushehr, Iran; 6 Transplant Research Center, Shiraz University of Medical Sciences, Shiraz, Iran; 7 School of Medicine, Shiraz University of Medical Sciences, Shiraz, Iran; Kazan State Medical University: Kazanskij gosudarstvennyj medicinskij universitet Ministerstva zdravoohranenia Rossijskoj Federacii, RUSSIAN FEDERATION

## Abstract

**Background:**

Multimorbidity, defined as the co-occurrence of multiple health conditions, is a major global public health concern. This study aimed to identify latent classes of multimorbidity and associated risk factors in Iranian adults.

**Method:**

This cross-sectional study analyzed baseline data from 10,131 adults who participated in the Fasa Adults Cohort Study (FACS) in southern Iran. Multimorbidity was defined as the presence of two or more of 11 chronic diseases, including hypertension, dyslipidemia, stroke, osteoarthritis, depression, type two diabetes mellitus, obesity, osteoporosis, cardiovascular disease, thyroid disease, and respiratory disease. Latent class analysis (LCA) was used for cluster participants, and multinomial logistic regression was conducted to investigate the association between age, sex, education level, socioeconomic status, daily sleep duration, physical activity, and multimorbidity.

**Result:**

The prevalence of multimorbidity was 40.3%. Three latent classes were identified: healthy (66.8%), dyslipidemia (14.1%), and cardio-metabolic conditions (19.1%). Older age increased the odds of belonging to dyslipidemia (odds ratio (OR) = 1.04 [95% confidence interval (CI): 1.03–1.05]) and cardio-metabolic conditions (OR = 1.10 [95% CI: 1.09–1.11]) classes. Similarly, women were at higher odds than men of being in dyslipidemia (OR = 2.49 [95% CI: 2.05–3.02]) and cardio-metabolic conditions (OR = 3.35, 95% CI: 2.79–4.03]) classes. Employed participants showed decreased odds of having cardio-metabolic conditions (OR = 0.66 [95% CI: 0.55–0.80]). However, very high socioeconomic status was a risk factor for cardio-metabolic conditions (OR = 1.44 [95% CI: 1.16–1.78]) and dyslipidemia (OR = 1.35 [95% CI: 1.10–1.65]). Higher physical activity and sleeping for 8 hours or more were protective factors against cardio-metabolic conditions (OR = 0.74 [95% CI: 0.63–0.87]). Moreover, medium or high dietary intake increased the odds of belonging to the dyslipidemia class (OR = 1.46 [95% CI: 1.09–1.94] and OR = 1.57 [95% CI: 1.16–2.11], respectively).

**Conclusion:**

Using LCA, we identified distinct subgroups of chronic diseases, showing hidden patterns of multimorbidity associated with several risk factors. This approach offers deeper knowledge of disease clustering, contributes to a more comprehensive understanding of multimorbidity, and shows the importance of regional health challenges in designing targeted public health interventions.

## Introduction

Multimorbidity, defined as the co-occurrence of multiple chronic health conditions, is emerging as a major public health concern, particularly in high-income countries [1]. However, surprisingly high prevalence rates of multimorbidity are also observed in low- and middle-income countries [2,3]. In Iran, the prevalence of multimorbidity is estimated to be 21.1%, with higher rates among older individuals and females [4]. Multimorbidity is associated with adverse health outcomes, including impaired physical and mental function, reduced quality of life, frailty, and increased mortality risk [5–8]. These effects may result from the synergistic accumulation of diseases rather than their individual impacts [9].

The coexistence of two or more chronic health conditions in an individual necessitates complex and structured medical care and increases healthcare utilization and financial burden [10,11]. Therefore, current single-disease-oriented public health and healthcare strategies are deemed inadequate, reflecting the need for a comprehensive understanding of multimorbidity patterns, and integrated treatment approaches [12]. To establish effective health policies and treatment plans for multimorbidity, it is essential to identify multimorbidity patterns in populations [13].

Cardio-metabolic-related disorders, such as hypertension, dyslipidemia, type two diabetes mellitus (T2DM), and stroke, which share pathophysiological pathways are commonly observed in multimorbid individuals [12,14,15]. Multimorbidity is a complex phenomenon that requires statistical approaches to group populations into subgroups with similar combinations of chronic diseases. Latent class analysis (LCA), a subset of structural equation modeling, is a useful method for identifying subgroups of individuals with common characteristics [16]. Using health-related data, this probabilistic modeling algorithm can explore the latent variables behind observed categorical variables that are statistically associated [17], to identify common combinations of chronic diseases in a population and help develop targeted interventions for specific subgroups of patients with multimorbidity [12]. Several studies have utilized LCA to identify multimorbidity patterns and associated factors [12,18–21]. For example, a study in South Africa identified four latent classes among the multimorbid population, with women comprising 70% of the population, and found that multimorbidity occurred across all age groups [20].

Noncommunicable diseases have a disproportionate distribution in societies, hence identifying subgroups using LCA in populations with shared characteristics can reflect the different healthcare priorities and needs of these societies [21]. Unlike previous studies that primarily assessed the effects of risk factors on individual chronic diseases or multimorbidity, this research employs LCA to uncover hidden patterns within chronic diseases. Given the potential of LCA in identifying disease patterns and associated factors, as well as the limited use of LCA for multimorbidity analysis in Iran, this study aimed to identify multimorbidity patterns and investigate their relationship with various risk factors using baseline cohort data from southern Iran.

## Materials and methods

### Setting and participants

The present cross-sectional study utilized baseline data from the Fasa Adult Cohort Study (FACS), located in Fars province, southern Iran. FACS is part of the larger PERSIAN cohort study, which aims to enroll 180,000 middle-aged Iranian adults (aged 35–70 years) from 18 distinct geographical regions in Iran. FACS is designed to investigate various demographic, socioeconomic, anthropometric, nutritional, lifestyle, and environmental factors associated with non-communicable diseases among 10,000 participants. The Central Iran Cohort Team has approved the validity and reliability of the questionnaires used in data collection. The cohort was established in 2015, and baseline data collection was completed by 18/10/2016. We accessed this data on 15/03/2022. The study will continue for a 15-year follow-up period [22]. For this cross-sectional study, we utilized the baseline cohort data. Each participant has an ID number, but we did not have any information to identify individuals.

### Measurement of included variables

The baseline data collection in FACS involved a comprehensive questionnaire comprising three main parts: general information, medical checklist, and nutrition assessment. The general information section included recording socioeconomic status, job history, daily living energy source and place of residence, lifestyle, sleep quality, physical activity, and biological samples. The medical checklist covered participants’ fertility history, chronic diseases, medications, family history, oral health, blood pressure, electrocardiogram, physical examination, physical disabilities, personal habits, and nutrition assessment [22]. Trained personnel administered these questionnaires through face-to-face interviews.

In this study, we extracted demographic variables such as age, sex, marital status (unmarried/married), education level (illiterate/ literate), occupation (unemployed/employed), socioeconomic status (low, moderate, or high), daily sleep duration (≤ 5, 5–6, or ≥ 8 hours), physical activity, and nutritional status. Sleep duration was extracted from the Pittsburgh Sleep Quality Index (PSQI) form, and physical activity was assessed using the Metabolic Equivalent Tasks (MET) as a proxy, calculated based on data from the International Physical Activity Questionnaire (IPAQ) [23]. Additionally, nutritional assessment was conducted using a modified 148-item food frequency questionnaire (FFQ) to capture eating habits and food consumption over the past year. This questionnaire, based on Willett’s format [24], was adapted to reflect Iranian food items. Participants were asked about their daily, weekly, monthly, and annual consumption of each listed food item, and food portion sizes were established based on the United States Department of Agriculture (USDA) measurements, then translated into grams per day. The corresponding variable we used for the nutritional assessment in the present study is called “dietary intake”, which is derived from previously published evidence on the FACS by our team. This variable poses three classes (or clusters) of low-, medium-, and high-intake profiles, which were obtained according to their dietary intake using latent profile analysis (LPA) [25].

Multimorbidity was defined as the presence of two or more out of 11 chronic conditions, including hypertension, dyslipidemia, stroke, osteoarthritis, depression, T2DM, obesity, osteoporosis, cardiovascular disease, thyroid disease, and respiratory disease [26]. The occurrence of this pattern was determined by locating participants who simultaneously had at least 2 out of the 11 chronic conditions specified. This prevalence (40.3%) was calculated from the total number of participants who provided complete information regarding their chronic disease status.

### Ethics approval

The PERSIAN Cohort Study, including the Fasa region, received approval from the Ethics Committees of the Ministry of Health and Medical Education. This study adheres to the principles outlined in the Helsinki Declaration and Iranian national guidelines for research ethics (Reference number: IR.FUMS.REC.1400.168). Informed written consent was obtained from all participants.

### Statistical analysis

Eligible participants in the study were selected from the FACS cohort to conduct Latent Class Analysis (LCA) analysis. The data were initially evaluated for incompleteness and outliers. Dichotomous variables were also used to perform this analysis and modeling. LCA was used to identify distinct latent classes based on 11 chronic diseases, which served as indicators of multimorbidity. LCA is a statistical technique that identifies subgroups within a population, where the subgroup memberships are not directly observed but inferred from the data. In our LCA model, the latent variable was categorical, which consisted of a set of indicator variables. The LCA models were estimated using maximum likelihood estimation, testing between 2–6 latent classes. The model selection process was based on fit indices including the Akaike Information Criterion (AIC), Bayesian Information Criterion (BIC), and the G² statistic. The AIC and BIC are widely used model selection criteria that balance model fit with complexity, penalizing models with more parameters to avoid overfitting. The BIC, in particular, is known to favor simpler models and has been shown to be the most accurate criterion for selecting the optimal number of latent classes in Monte Carlo simulations. A model with the lowest AIC and BIC values was selected as the best-fitting model [12]. Each participant was assigned to the latent class with the highest predicted probability of membership. Following this, multinomial logistic regression was used to examine the association between multimorbidity latent class membership and studied risk factors, reporting odds ratios (OR) with 95% confidence intervals (CI).

Descriptive statistics, including mean with standard deviation (SD) and count with percentage, were used to report continuous and categorical variables, respectively. The Chi-square test and ANOVA were used to assess univariable associations between categorical and continuous variables, respectively, and multimorbidity classes. All analyses were performed using poLCA in the R (version 4.1.1) software environment [27].

## Results

**Fig 1:**
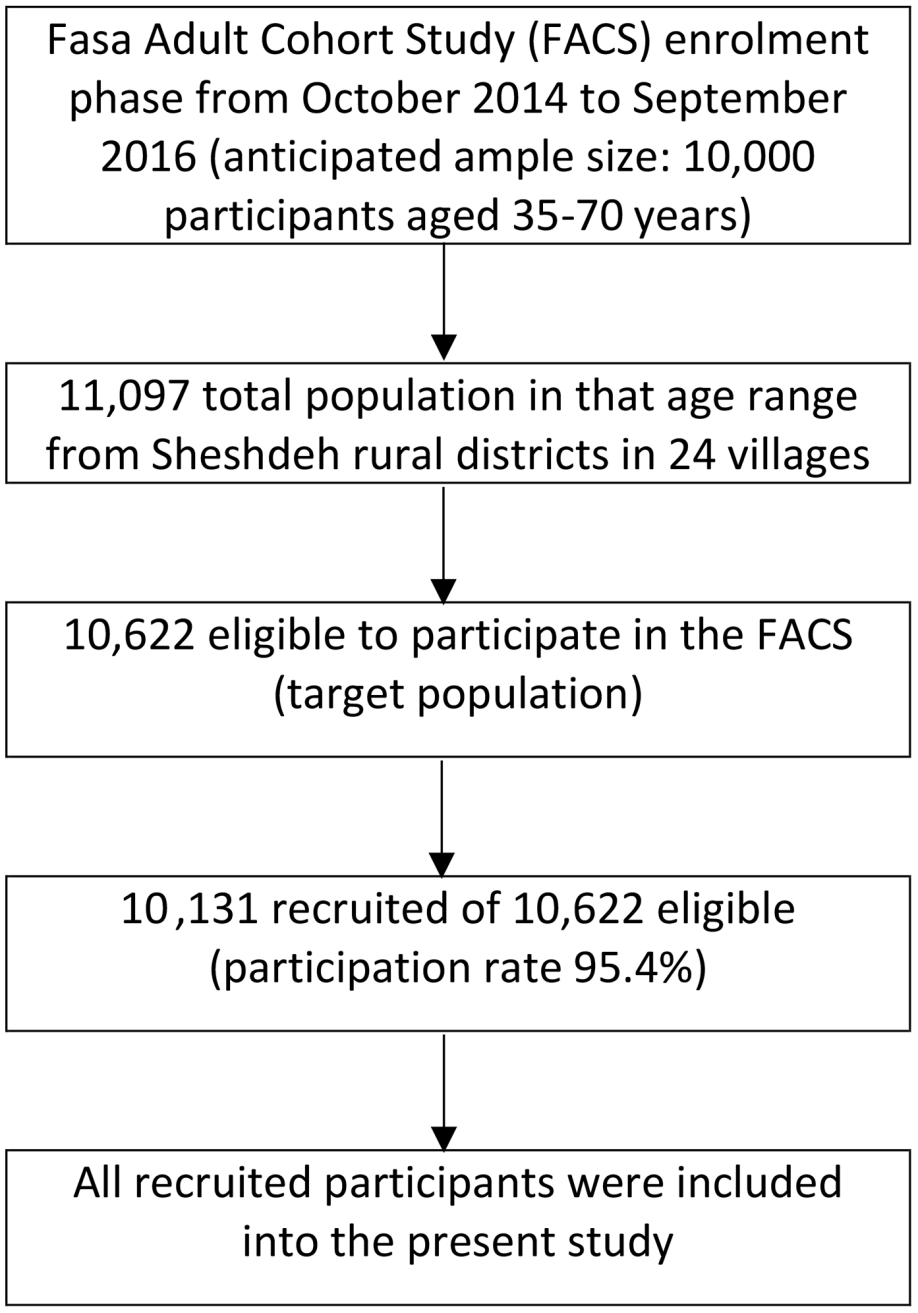
Flow diagram of the included participants. (A) Between October 2014 and September 2016, a total of 11,097 people aged 35 to 70 years from 24 villages in the Sheshdeh rural area were evaluated. (B) Out of these, 10,622 qualified, and 10,131 were enrolled (participation rate: 95.4%). (C) All participants who were recruited were incorporated into this study.

A total of 10,131 participants were included in the study, with a mean age of 48.64 ± 9.58 years, and the majority were women (54.8%). The flow diagram of the included participants is depicted in Fig 1. Among the participants, 54.2% were literate, 50.5% were employed, and approximately 88% were married. The prevalence of multimorbidity was 40.3%, with multimorbid individuals having a mean of 2.8 chronic conditions. Dyslipidemia (45.1%), hypertension (20.1%), and T2DM (19.7%) were the most prevalent chronic conditions. Table 1 summarizes detailed descriptive information about the study participants.

**Table 1:** Descriptive information of the FACS participants.

Frequency (%)	Variable
Age, *year* (mean ± SD)	48.64 ± 9.58
Sex	
Male	4575 (45.2)
Female	5556 (54.8)
Educational level	
Illiterate	4641 (45.8)
Literate	5490 (54.2)
Marital status	
Unmarried	1120 (11.1)
Married	9011 (88.9)
Occupation	
Unemployed	5016 (49.5)
Employed	5115 (50.5)
Socioeconomic status	
Low	2533 (25.0)
Moderate	2534 (25.0)
High	2532 (25.0)
Very high	2532 (25.0)
Daily sleep duration, *hour per day*	
≤ 5	2082 (22.4)
6–7	1886 (20.3)
≥ 8	5334 (57.3)
Dietary intake	
Low intake	740 (7.3)
Medium intake	3837 (37.9)
High intake	3684 (36.4)
MET (mean ± SD)	41.41 ± 11.45
Prevalence of comorbid conditions	
Hypertension	2032 (20.1)
Dyslipidemia	4567 (45.1)
Stroke	129 (1.3)
Osteoarthritis	348 (3.4)
Depression	685 (6.8)
Diabetes mellitus	1995 (19.7)
Thyroid disease	890 (8.8)
Obesity	1795 (17.7)
Cardiovascular disease	1103 (10.9)
Osteoporosis	993 (9.8)
Lung disease	192 (1.9)
Multimorbidity ^2^	4080 (40.3)
Number of co-occurring diseases (mean ± SD)	2.81 ± 1.04

^1^ ≥ 2 chronic conditions.

Abbreviations: MET, Metabolic Equivalent of Task.

LCA was performed with models ranging from 2 to 6 classes. The model selection criteria, including G^2^, AIC, and BIC, were used to determine the best-fitting model. Due to the magnitude of the G^2^ reference statistics and the high number of estimated parameters, the distribution of this index was unknown; therefore, the role of AIC and BIC in selecting the model was more pronounced – the best model is the one with the lowest values. The three-class model had the lowest BIC value, while the four-class model had the lowest AIC value. However, for better interpretability, the three-class model was selected as the best fit. The performance indicators of the models are shown in table 2.

**Table 2:** Model selection statistics from LCA models with different latent classes.

Number of classes	Number of parameters estimated	G^2^	df	AIC	BIC	X^2^	Maximum log-likelihood
2	23	1364	2024	72094	72260	16947	−35934.07
3	35	1184	2012	72094	72260	10179	−35934.07
4	47	1081	2000	71859	72198	11387	−35882.53
5	68	1018	1988	151.59	484.49	9572	−35850.98
6	71	941	1976	172.13	573.58	2598	−35812.70

Abbreviations: LCA, latent class analysis; AIC, Akaike information criterion; BIC, Bayesian information criterion.

**Fig 2:**
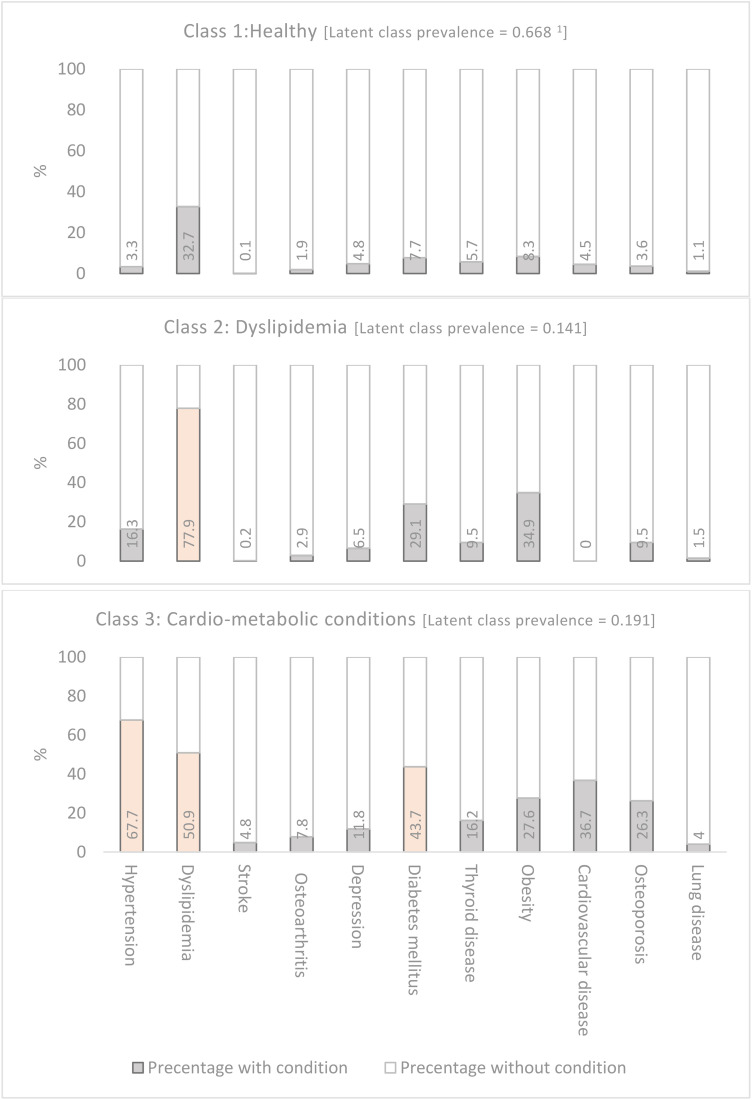
Item response probability for multimorbid conditions across each multimorbidity cluster. (A) Class 1 (Healthy; prevalence = 0.668), Class 2 (Dyslipidemia; prevalence = 0.141), and Class 3 (Cardio-metabolic conditions; prevalence = 0.191) notes: ^1^ The probability of a “No” response can be calculated by subtracting the item-response probabilities from 1. ^2^ Item-response probabilities >0.5 are colored light orange to facilitate interpretation.

The probability of item responses for multimorbid conditions across each multimorbidity cluster is shown in Fig 2. Participants were assigned to one of three classes based on the highest membership probability. The healthy class had a prevalence of 66.8% and low probabilities of all studied chronic diseases. The dyslipidemia class, with a prevalence of 14.1%, had a high probability of dyslipidemia (77.9%). The cardio-metabolic class, with a prevalence of 19.1%, had high probabilities of hypertension (67.7%), T2DM (43.7%), and dyslipidemia (50.9%).

The cardio-metabolic class had the highest mean age (55.36 ± 8.75 years), with 53.7% of men and 46.3% of women classified as healthy. Women were significantly more likely than men to belong to the dyslipidemia and cardio-metabolic classes (P < 0.001). Illiteracy, low socioeconomic status, unemployment, and unmarried status were significantly higher among cardio-metabolic class participants compared to the other two classes (P < 0.001 for all). Additionally, more than 59% of healthy class participants slept 8 hours or more per day, while cardio-metabolic class participants had significantly lower daily sleep hours and physical activity index (P < 0.001). The mean number of co-occurring diseases was significantly higher in the cardio-metabolic and dyslipidemia classes compared to the healthy class (P < 0.001) (Table 3).

**Table 3:** Comparisons of demographic and clinical data among participants by latent class membership.

Variable	Latent Classes			P
	Healthy	Dyslipidemia	Cardio-metabolic conditions	
Age, *year* (mean ± SD)	46.59 ± 8.96	46.59 ± 8.96	55.36 ± 8.75	
Sex				
Male	3640 (53.7)	488 (34.1)	447 (23.2)	<0.001 ^‡^
Female	3134 (46.3)	944 (65.9)	1478 (76.8)	
Marital status				
Unmarried	655 (9.7)	151 (10.5)	314 (16.3)	<0.001 ^‡^
Married	6119 (90.3)	1281 (89.5)	1611 (83.7)	
Occupation				
Unemployed	2760 (40.7)	835 (58.3)	1421 (73.8)	<0.001 ^‡^
Employed	4014 (59.3)	597 (41.7)	504 (26.2)	
Education level				
Illiterate	2629 (38.8)	671 (46.9)	1341 (69.7)	<0.001 ^‡^
Literate	4145 (61.2)	761 (53.1)	584 (30.3)	
Socioeconomic status				
Low	1625 (24.0)	311 (21.7)	597 (31.0)	
Moderate	1708 (25.2)	330 (23.0)	496 (25.8)	<0.001 ^‡^
High	1684 (24.9)	406 (28.4)	442 (23.0)	
Very high	1757(25.9)	385 (26.9)	390 (20.3)	
Daily sleep duration, *hour per day*				
≤ 5	1279 (20.6)	292 (22.3)	511 (28.5)	
6–7	1258 (20.3)	255 (19.5)	373 (20.8)	<0.001 ^‡^
≥ 8	3666 (59.1)	760 (58.1)	908 (50.7)	
Dietary intake				
Low intake	69 (6.2)	233 (14.6)	438 (7.9)	
Medium intake	513 (46.2)	835 (52.2)	2489 (44.8)	<0.001 ^‡^
High intake	528 (47.6)	532 (33.3)	2624 (47.3)	
MET (mean ± SD)	42.74 ± 12.26	39.40 ± 9.40	38.21 ± 8.74	<0.001 ^†^
Number of co-occurring diseases	2.05 ± 0.22	2.37 ± 0.58	3.42 ± 1.14	<0.001 ^†^

† ANOVA

‡ Chi-square test

Abbreviations: MET, Metabolic Equivalent of Task.

A multinomial logistic regression model was developed, adjusting for age, sex, education level, occupation, marital status, socioeconomic status, physical activity, and sleep duration. Results showed that with every year increase in age, the odds of belonging to dyslipidemia (odds ratio (OR) = 1.04, 95% CI: 1.03–1.05, P < 0.001) and cardio-metabolic conditions (OR = 1.10, 95% CI: 1.09–1.11, P < 0.001) classes significantly increased compared to the healthy class. Women had significantly higher odds than men of belonging to dyslipidemia (OR = 2.49, 95% CI: 2.05–3.02, P < 0.001) and cardio-metabolic (OR = 3.35, 95% CI: 2.79–4.03, P < 0.001) classes. Eemployed status was a protective factor against belonging to the cardio-metabolic conditions class (OR = 0.66, 95% CI: 0.55–0.80, P < 0.001). Conversely, very high socioeconomic status increased the odds of belonging to both dyslipidemia (OR =1.44, 95% CI: 1.16–1.78, P = 0.001) and cardio-metabolic conditions (OR = 1.35, 95% CI: 1.10–1.65, P = 0.003) classes. Additionally, sleeping for 8 hours or more per day (OR = 0.74, 95% CI: 0.63–0.87, P < 0.001) was associated with reduced odds of membership in the cardio-metabolic conditions class. Moreover, every unit increase in MET decreased the odds of membership in dyslipidemia classes (OR = 0.98, 95% CI: 0.97–0.98, P < 0.001) and cardio-metabolic conditions (OR = 0.97, 95% CI: 0.96–0.98, P < 0.001). Furthermore, the high dietary intake group had higher odds of belonging to the dyslipidemia class (OR = 1.57, 95% CI: 1.16–2.11, P = 0.003) compared to the healthy class (Table 4).

**Table 4:** Summary findings of multinomial logistic regression of association between multimorbidity and studied risk factors.

Variable	Latent Classes [Odds Ratio (95% CI)]		
	**Healthy**	**Dyslipidemia**	**Cardio-metabolic conditions**
Age, *year*	1	**1.04 (1.03 - 1.05)**	**1.10 (1.09 - 1.11)**
Sex			
Male	1	1	1
Female	1	**2.49 (2.05–3.02)**	**3.35 (2.79–4.03)**
Marital status			
Unmarried	1	1	1
Married	1	1.17 (0.93 - 1.48)	1.12 (0.91–1.37)
Occupation			
Unemployed	1	1	1
Employed	1	1.12 (0.92- 1.36)	**0.66 (0.55 - 0.80)**
Education level			
Illiterate	1	1	1
Literate	1	1.04 (0.88 - 1.24)	0.87 (0.74–1.03)
Socioeconomic status			
Low	1	1	1
Moderate	1	1.16 (0.94–1.42)	1.15 (0.95 - 1.37)
High	1	**1.37 (1.12 - 1.96)**	1.18 (0.98 - 1.43
Very high	1	**1.44 (1.16 - 1.78)**	**1.35 (1.10 - 1.65)**
Daily sleep duration, *hour per day*			
≤ 5	1	1	1
6–7	1	0.93 (0.75 - 1.16)	0.86 (0.71 - 1.05)
≥ 8	1	0.99 (0.83 - 1.18)	**0.74 (0.63 - 0.87)**
Dietary intake			
Low intake	1	1	1
Medium intake	1	**1.46 (1.09–1.94)**	1.04 (0.84–1.29)
High intake	1	**1.57 (1.16–2.11)**	0.90 (0.71–1.13)
MET	1	0.98 (0.97 - 0.098)	**0.97 (0.96 - 0.98)**

Abbreviations: MET, Metabolic Equivalent of Task; CI, confidence interval.

Bold ORs with 95% CIs represent statistically meaningful.

## Discussion

The primary aim of this study was to identify distinct patterns of multimorbidity in the adult population of southern Iran, with an emphasis on understanding how multiple chronic conditions co-occur, rather than examining each condition separately. Using latent class analysis (LCA), we explored the clustering of chronic diseases within individuals and investigated the socio-demographic and lifestyle factors associated with these patterns. The present study identified three distinct classes: a relatively healthy class, a dyslipidemia class, and a cardio-metabolic disorders class encompassing hypertension, T2DM, and dyslipidemia. This pattern-based approach provides a more integrated understanding of multimorbidity, highlighting disease combinations that may share common etiological pathways and require targeted prevention and management strategies. Comparing our findings with previous studies is challenging due to variations in study environments, definitions, disease spectra, demographic factors, data collection methods, and participants’ baseline health statuses [28–31]. Nonetheless, we attempted to compare our identified disease patterns with those reported in prior literature.

Our study estimated the prevalence of multimorbidity in the general population of southern Iran to be 40.3%, with LCA identifying rates of 14.1% for dyslipidemia and 19% for cardiometabolic classes. For comparison, a meta-analysis of 37 studies reported global multimorbidity prevalence ranging from 3.5% to 70%. Another systematic review involving 15.4 million adults found an overall prevalence of 37.2%, with notable regional variations: South America (45.7%), North America (43.1%), Europe (39.2%), and Asia (35%). Our findings align with similar studies conducted in Asia but are slightly higher than those reported for Europe and North America. These results highlight significant regional and demographic differences in the prevalence of multimorbidity. It is important to note that although most studies applied a similar general definition of multimorbidity (i.e., the coexistence of two or more chronic conditions), the number and type of conditions considered varied substantially across studies, ranging from a few to several dozen diseases. Despite this heterogeneity, hypertension, diabetes, dyslipidemia, and stroke were among the most frequently included conditions. These are largely consistent with the disease spectrum considered in our study, which enhances the comparability of our findings. However, differences in disease lists and data sources across studies may partly explain the variability observed in multimorbidity prevalence estimates. This variability underscores the importance of conducting region-specific investigations, such as ours, to better understand local patterns and inform targeted healthcare interventions [32,33]. Additionally, our study identified a high likelihood of co-occurrence among dyslipidemia, hypertension, and T2DM, consistent with findings from other studies. For instance, Fortin et al. reported hypertension, hyperlipidemia, and rheumatological diseases as common disorders in multimorbidity, while another study found hypertension, hyperlipidemia, and T2DM among 45 chronic diseases [34,35].

The regression analysis demonstrated that older age and female sex were strongly associated with belonging to multimorbidity classes. This finding is consistent with previous research and may be explained by age-related accumulation of chronic conditions and women’s longer life expectancy, biological differences, and greater healthcare utilization. Interestingly, very high socioeconomic status was also associated with increased multimorbidity risk, which may reflect lifestyle-related factors such as dietary habits, stress, and sedentary behavior observed in affluent populations. Conversely, protective factors such as employment, higher physical activity, and adequate sleep suggest that social engagement, healthy lifestyles, and restorative behaviors play an important role in mitigating multimorbidity. These results emphasize the need for integrated prevention strategies focusing not only on biological determinants but also on modifiable behavioral and social factors [12,36,37].

Women had a significantly higher likelihood of belonging to dyslipidemia and cardio-metabolic classes compared to men, aligning with other studies indicating a higher prevalence of multimorbidity among women [19,38,39]. In contrast to our study, Petty et al. found no association between sex and the higher risk of multimorbidity [40]. This gender disparity in multimorbidity prevalence may be attributed to factors such as longer lifespan, biological differences, and socio-cultural factors [38,41].

Our study also showed an association between unemployment and increased odds of belonging to cardio-metabolic classes. Although information on this association is scarce, some evidence might be consistent with ours. That is, people with low levels of education may have limited health literacy, low socioeconomic status, lower chance of employment, and reduced access to healthcare resources; therefore, they may have poor management in preventing and controlling disease risk factors. A study of Danish adults found that low education greatly increased the likelihood of belonging to class 6 (Complex Cardio-metabolic Disorders) and class 7 (Complex Respiratory Disorders) of multimorbidity [19]. Other studies also have shown that a low level of education is consistently associated with an increased risk of multimorbidity [42–44].

Longer sleep duration (>8 hours per day) was inversely associated with membership in the cardio-metabolic class. This finding is consistent with studies linking short sleep duration to increased mortality risk in individuals with chronic diseases such as T2DM, hypertension, cardiovascular disease, and obesity [45,46]. In a large cohort study conducted in the United States, a U-shaped relationship was reported between sleep duration and all-cause and cardiovascular mortality compared to normal sleep; that is, sleeping less than 5 hours and more than 9 hours increased total mortality by 16% and 11%, respectively [46]. These findings might highlight the importance of adequate sleep-in disease prevention and management.

Additionally, higher levels of physical activity, measured in metabolic equivalent of task (MET) units, were associated with a reduced likelihood of membership in lipid disorders and cardiometabolic classes, consistent with findings from previous studies [47]. However, a study by Autenrieth et al. observed no link between physical activity and multimorbidity in women, while Hudon et al. found no such relationship in either sex after adjusting for age, education, income, and employment. Both studies highlighted limitations, including reliance on self-reported data for physical activity and chronic diseases, as well as potential recall bias [48,49]. Beyond these methodological limitations, variations in the type and number of comorbidities examined may also account for these discrepancies. Nonetheless, with the aging global population and the rising prevalence of multimorbidity, our findings underscore the importance of physical activity as a cornerstone of prevention and health promotion efforts [50,51].

Moreover, our results also showed the association between dietary intake and multimorbidity, particularly with dyslipidemia. Medium or high intake levels were found to increase the odds of belonging to the dyslipidemia class. The importance of diet in multimorbidity prevention has been widely recognized. For example, a study of 35,372 participants in the UK cohort found that higher daily energy intake was associated with an 8% increased risk of multimorbidity (HR: 1.08 (95% CI: 1.01, 1.16)) [52]. Therefore, it might represent the significance of promoting healthy dietary patterns as a strategy for mitigating multimorbidity risks.

In addition, the association between very high socioeconomic status and increased multimorbidity risk underscores the need to explore lifestyle factors and stressors in affluent populations. These findings emphasize the importance of a holistic, multi-level approach to reducing the burden of multimorbidity and improving the quality of life for vulnerable groups.

The findings of this study had significant public health implications. The high odds of multimorbidity among women and older adults represent the need for targeted interventions. For women, preventive care and chronic disease management programs are essential, as they are at higher risk for multiple conditions. For older adults, integrating multimorbidity management into primary care systems is critical to improve health outcomes. Moreover, the association between very high socioeconomic status and increased multimorbidity risk underscores the need to explore lifestyle factors and stressors in affluent populations. Additionally, employed participants showed decreased odds of experiencing cardio-metabolic conditions, and higher physical activity along with adequate sleep were identified as protective factors. Thus, promoting physical activity, healthy sleep habits, and addressing the risk factors associated with socioeconomic disparities are essential components of an effective public health strategy. Finally, our results indicate that medium or high dietary intake increased the odds of belonging to the dyslipidemia class, emphasizing the importance of nutrition interventions in reducing the burden of chronic diseases. These findings suggest that public health policies should focus on both preventive measures and the management of multimorbidity in vulnerable groups.

A notable strength of this study is the use of LCA, which allowed us to identify distinct subgroups of multimorbidity based on 11 chronic conditions. However, the study has several limitations. First, the use of multinomial logistic regression with class membership as the outcome variable, derived from probabilistic LCA, might not fully account for the inherent uncertainty in class assignment, which represents a methodological consideration. Longitudinal studies are recommended in the future, particularly when follow-up data from the FACS become available. Second, despite performing quality control at FACS, reliance on self-reported baseline data introduces the possibility of reporting bias, particularly recall errors, which warrants caution when interpreting the results. Third, the findings of this study are based on a cohort of adults aged 35 years and older residing in a rural district of Fasa, Iran. This specific geographic focus limits the generalizability of our results to other regions, particularly urban areas or populations with different socio-economic or cultural characteristics. Furthermore, the demographic and health profiles of the Fasa cohort may not fully represent the broader Iranian population or other middle-income countries. Therefore, the external validity of the study findings may be restricted. Fourth, other unmeasured chronic diseases or risk factors were not included in the analysis, which could introduce residual confounding. Finally, the available data does not allow us to assess the impact of multimorbidity on long-term outcomes such as mortality, which could provide further insights into the burden of chronic diseases.

## Conclusion

This study aimed to identify multimorbidity patterns and their associated risk factors among adults in southern Iran using latent class analysis. We identified three distinct classes of multimorbidity: a relatively healthy group, a dyslipidemia group, and a cardio-metabolic group, with an overall multimorbidity prevalence of 40.3%. Our findings indicate that older age, female sex, very high socioeconomic status, and higher dietary intake were significantly associated with increased risk of belonging to multimorbidity classes, while employment, greater physical activity, and adequate sleep were protective factors. These results highlight the heterogeneous nature of multimorbidity and emphasize the role of both demographic and lifestyle determinants in shaping disease patterns. Recognizing such patterns is crucial for developing targeted prevention and management strategies, particularly for vulnerable subgroups such as women and older adults. Future longitudinal research using cohort follow-up data will be essential to confirm these associations, explore causal pathways, and guide the design of comprehensive, population-specific interventions to reduce the burden of multimorbidity.
